# Minichromosome maintenance 3 promotes hepatocellular carcinoma radioresistance by activating the NF-κB pathway

**DOI:** 10.1186/s13046-019-1241-9

**Published:** 2019-06-17

**Authors:** Wei Meng, Binhui Xie, Qing Yang, Changchang Jia, Hui Tang, Xiaomei Zhang, Yi Zhang, Jianwen Zhang, Heping Li, Binsheng Fu

**Affiliations:** 1 Department of Hepatic Surgery and Liver transplantation Center of the Third Affiliated Hospital, Organ Transplantation Institute, Sun Yat-sen University; Organ Transplantation Research Center of Guangdong Province, 600# Tianhe Road, Guangzhou, 510630 China; 2 Department of Hepatobiliary Surgery, The First Affiliated Hospital of Gannan Medical University, Ganzhou, 341000 China; 3 Cell-gene Therapy Translational Medicine Research Center, The Third Affiliated Hospital of Sun Yat-sen University, Guangzhou, 510630 China; 4 Guangdong Key Laboratory of Liver Disease Research, The Third Affiliated Hospital of Sun Yat-sen University, Guangzhou, 510630 China; 5 Department of Medical Oncology of the Eastern Hospital, The First Affiliated Hospital of Sun Yat-sen University, Zhongshan Er Road, Guangzhou, 510080 China

**Keywords:** MCM3, HCC, Radiotherapy resistance, NF-κB pathway

## Abstract

**Background:**

Hepatocellular carcinoma (HCC) is the most common tumors in the worldwide, it develops resistance to radiotherapy during treatment, understanding the regulatory mechanisms of radioresistance generation is the urgent need for HCC therapy.

**Methods:**

qRT-PCR, western blot and immunohistochemistry were used to examine MCM3 expression. MTT assay, colony formation assay, terminal deoxynucleotidyl transferase nick end labeling assay and In vivo xenograft assay were used to determine the effect of MCM3 on radioresistance**.** Gene set enrichment analysis, luciferase reporter assay, western blot and qRT-PCR were used to examine the effect of MCM3 on NF-κB pathway.

**Results:**

We found DNA replication initiation protein Minichromosome Maintenance 3 (MCM3) was upregulated in HCC tissues and cells, patients with high MCM3 expression had poor outcome, it was an independent prognostic factor for HCC. Cells with high MCM3 expression or MCM3 overexpression increased the radioresistance determined by MTT assay, colony formation assay, TUNEL assay and orthotopic transplantation mouse model, while cells with low MCM3 expression or MCM3 knockdown reduced the radioresistance. Mechanism analysis showed MCM3 activated NF-κB pathway, characterized by increasing the nuclear translocation of p65, the expression of the downstream genes NF-κB pathway and the phosphorylation of IKK-β and IκBα. Inhibition of NF-κB in MCM3 overexpressing cells using small molecular inhibitor reduced the radioresistance, suggesting MCM3 increased radioresistance through activating NF-κB pathway. Moreover, we found MCM3 expression positively correlated with NF-κB pathway in clinic.

**Conclusions:**

Our findings revealed that MCM3 promoted radioresistance through activating NF-κB pathway, strengthening the role of MCM subunits in the tumor progression and providing a new target for HCC therapy.

**Electronic supplementary material:**

The online version of this article (10.1186/s13046-019-1241-9) contains supplementary material, which is available to authorized users.

## Background

HCC is the fifth most common tumors worldwide [1]. Although the greatly improved in the last decades, its 5-year survival rate is only 15%, owing to the limitation of surgical intervention, radiotherapy and chemotherapy. It’s urgent need to identify potential biomarkers for prognosis and find new targets for designing more powerful therapeutic approach [2–4].

Eukaryotic DNA replication initiation includes helicase loading, helicase activation, replisome assembly and DNA synthesis, MCM2–7 complex assembled by six MCM subunits participates in all the events of DNA replication initiation [5–8]. Some subunits have been studied in HCC, for example, MCM7 is a poor prognostic factor for HCC and promotes HCC growth through activating MAPK signaling [9], MCM6 is a novel serum biomarker for early HCC and promotes HCC metastasis through activating MEK/ERK pathway [10]. MCM3 belongs to MCM2–7 complex, it is a poor prognosis marker for oral squamous cell carcinoma, melanoma, papillary thyroid carcinoma, cutaneous T-cell lymphomas, osteosarcoma, glioma, keratocystic odontogenic tumor, anaplastic astrocytoma and salivary gland epithelial tumors [11–20]. MCM3 is upregulated in prostate cancer tissues samples with bone metastasis, mouse model showed that MCM3 is increased in mesenchymal-derived tumors [21]. MCM3 also is upregulated in medulloblastoma and promotes cell migration and invasion [22]. But these studies only investigate whether MCM3 could be a prognostic factor for various tumors, its role in tumor progression couldn’t be well investigated. Especially, it’s role in radioresistance of HCC. In this study, we main studied the effect of MCM3 on radioresistance of HCC and its regulatory mechanism, we found MCM3 was an independent prognostic factor for HCC and promoted radiotherapy resistance through activating NF-κB pathway.

## Materials and methods

### Cell cultures

Immortalized normal liver cell LO2 and human HCC cell lines including SK-Hep1, SNU-475, HepG2, Huh7, Huh1, SNU-182 and Hep3B were purchased from the ATCC and cultured in DMEM high glucose (Hyclone) supplemented with 10% fetal bovine serum (FBS), the cells were maintained at 37 °C in 5% CO_2_ incubator.

### Tissues samples and immunohistochemistry (IHC)

Eighteen fresh tissue specimens of HCC and three fresh tissue of non-tumor adjacent tissue, as well as 162 paraffin-embedded HCC specimens were utilized, the detailed information was shown in Additional file 1: Table S1 The criteria for determining patient recurrence is that tumors is found in the liver, lung, skeleton, lymph and other positions after complete healing. These samples were collected during surgical procedures from patients with HCC according to a protocol approved by the institutional review board of the First Affiliated Hospital of Sun Yat-sen University. All patients provided written, informed consent for participation in the study and provision of tumor samples. IHC was performed according to our previous methods [23, 24]. Anti-MCM3 antibody (ab4460, Abcam) was used. The images were captured using the AxioVision Rel.4.6 computerized image analysis system (Carl Zeiss Co Ltd., Jena, Germany).

### Vectors, lentiviral infection and transfection

Human MCM3 cDNA was subcloned into the pSin-EF1α-puro lentiviral vector to generate pSin-EF1α-MCM3 vector (indicated as MCM3), the empty vector was used as the negative control (indicated as Vector). Two short hairpin RNAs (shRNAs) oligonucleotides sequences against MCM3 was cloned into the PLKO.1 lentiviral vector to generate PLKO.1-MCM3 shRNAs (indicated as shRNA#1 and shRNA#2, respectively), The sequences of shRNAs were: shRNA#1, 5′ GCCACAGATGATCCCAACTTT3’ and shRNA#2, 5′ GCAGGATGACAATCAGGTCAT3’. the scramble shRNA sequence was cloned PLKO.1 vector and used as the negative control (indicated as Scramble). These vectors were cotransfected with pM2.G and psPAX2 into 293 T using Exfect Transfection Reagent (Vazyme, Nanjing, China). The lentiviral supernatants were collected 48 h after transfection and filtered through a 0.45 μm filter. Supernatants plus polybrene (Sigma) were infected with growing HCC cells, after 12 h the supernatants were replaced by fresh medium. Puromycin (Sigma) was used to screen stably cell lines.

### Radiation treatment

HCC cells were irradiated by different radioactive rays Gy (0.5, 1.0, 1.5, 2.0, 2.5 and 3.0) from 6Mv-X-ray produced by a linear accelerator (Varian 600, Varian Medical Systems). The following day after irradiation, cells were used as MTT assy. Cells treated with 2 Gy radioactive rays were used as colony formation assay and TUNEL assay.

### Cell proliferation assay

MTT assay, colony formation assay and terminal deoxynucleotidyl transferase nick end labeling (TUNEL) assay were performed according to our previous methods [25–27].

### qRT-PCR

Total RNA was extracted using RNA isolater Total RNA Extraction Reagent (Vazyme), and reversely transcribed into cDNA using HiScript II 1st Strand cDNA Synthesis Kit with gDNA wiper (Vazyme). Relative gene expression levels were examined using AceQ qPCR SYBR Green Master Mix (Vazyme) on a CFX96 Touch Real-time PCR Detection system (Bio-Rad). GAPDH was used as the internal control.

### Western blot

Total proteins were extracted using RIPA buffer (50 mM Tris (pH 7.4), 1 mM EDTA, 150 mM NaCl, 1% NP-40, 0.5% sodium deoxycholate) supplemental with protease inhibitors (Roche). KeyGEN Nuclear and Cytoplasmic Protein Extraction Kit (KGP150, KeyGEN BioTECH) was used to isolate nuclear proteins. Antibodies against MCM3 (ab4460, Abcam), p65 (ab16502, Abcam), p84 (ab487, Abcam), IKKβ (ab124957, Abcam), p-IKKβ (ab38515, Abcam), IκBα (ab32518, Abcam), p-IκBα (ab133462, Abcam), DNA PKcs (ab32566), DNA PKcs (phosphor S2056) (ab18192), CLEAVED PARP1 (ab32064) and GAPDH (G8795, Sigma).

### In vivo xenograft assay

All animal experiments were performed under the protocols approved by the Institutional Animal Care and Use Committee of the First Affiliated Hospital of Sun Yat-sen University. Six weeks old BALB/c-nu mice were purchased from the Experimental Animal Center of the Guangzhou University of Chinese Medicine. 5◊10^6^ HepG2 with MCM3 overexpression or knockdown were orthotopically injected into the liver parenchyma of mice (*n* = 6) to observe the tumor growth, tumor size was up to 7.0–8.0 mm, the mice were treated with 10Gy radioactive rays. The mice were continued to feed for 40 days, then were euthanized, tumors were excised.

### Statistical analysis

SPSS 19.0 was used to perform all statistical analyses. All data from at least three independent experiments are presented as the mean ± s.d. Comparisons between different groups were analyzed using Student’s *t*-test, Survival curves were derived from Kaplan-Meier estimates, multivariate Cox-regression analysis was used to determine the prognostic value of MCM3 levels and other clinicopathologic characteristics. RNA-seq data from the TCGA HCC data set portal were used for the analyzing MCM3 expression, Salmon and DESeq2 were used to analyze MCM3 expression in HCC samples and normal liver samples. Gene set enrichment analysis (GSEA) were performed using GSEA 2.0.9 software http://software.broadinstitute.org/gsea/index.jsp. *p* < 0.05 was considered to be statistically significant.

## Results

### High MCM3 expression is associated with poor outcome for HCC patients

**Fig. 1:**
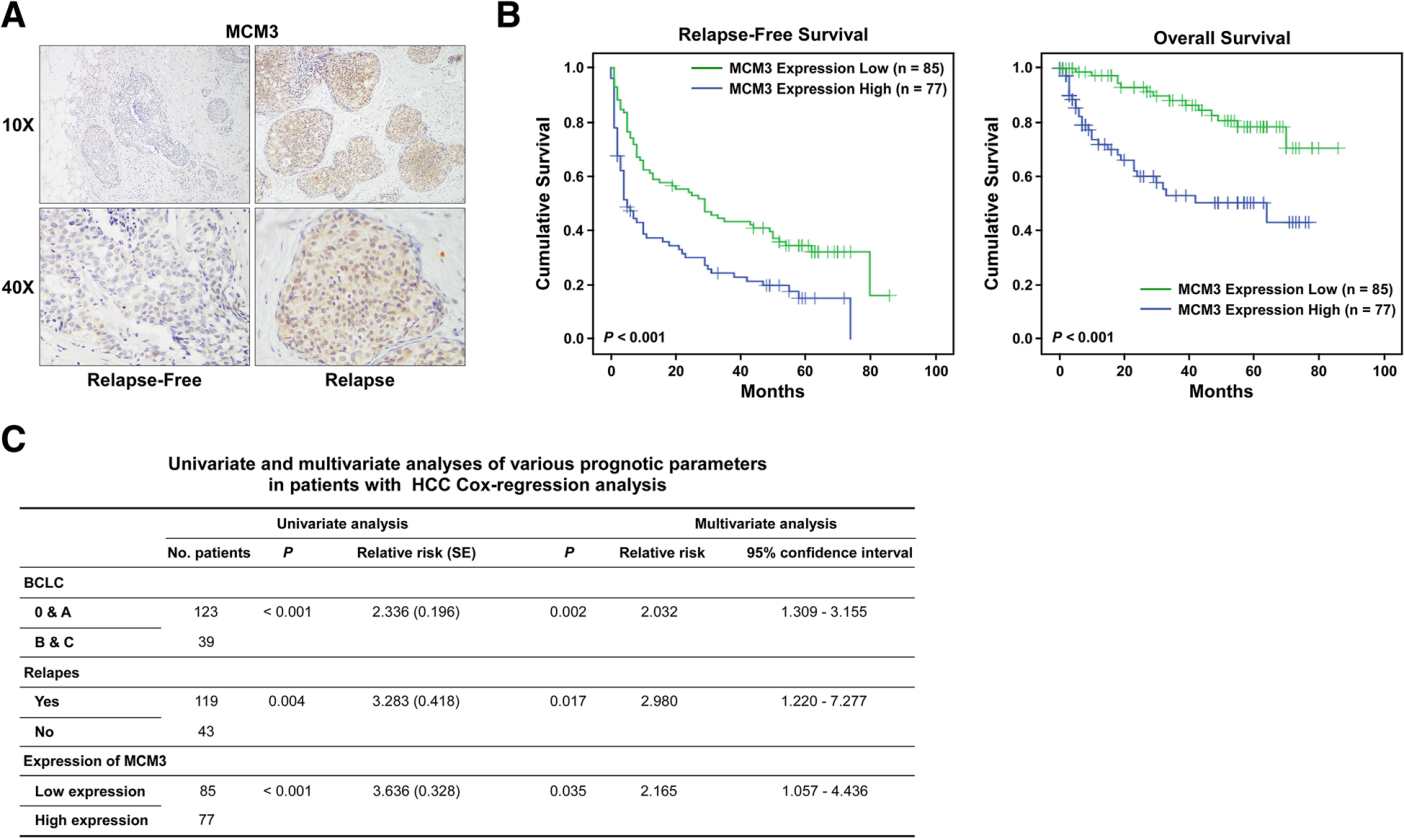
MCM3 is an independent prognostic factor for HCC. **a** IHC images indicated MCM3 expression in relapse-free HCC tissues and relapse HCC tissues. **b** Kaplan-Meier analysis of relapse-free and overall survival curves of patients with high MCM3 expression versus low MCM3 expression. **c** Multivariate Cox regression analysis to investigate the importance of MCM3 in clinical prognosis

To determine the role of MCM3 in HCC progression, we determined MCM3 level in HCC tissues with relapse or without relapse using IHC and found MCM3 was upregulated in tissues with relapse compared to tissues without relapse (Fig. 1a). We performed a Kaplan-Meier analysis to determine the relationship between MCM3 expression and the survival of patients, patients with high MCM3 expression had shorter survival time compared to patients with high MCM3 expression for relapse-free survival and overall survival (Fig. 1b). We also investigated whether MCM3 could serve as an independent prognostic factor, univariate analysis showed that clinical stage, relapse and MCM3 expression were associated with patients’ survival time. Multivariate analysis showed clinical stage, relapse and MCM3 expression also were independent prognostic factors for patients’ survival time (Fig. 1c). These results showed that MCM3 was a poor prognostic factor for HCC patients.

### MCM3 is upregulated in HCC cells and tissues

**Fig. 2:**
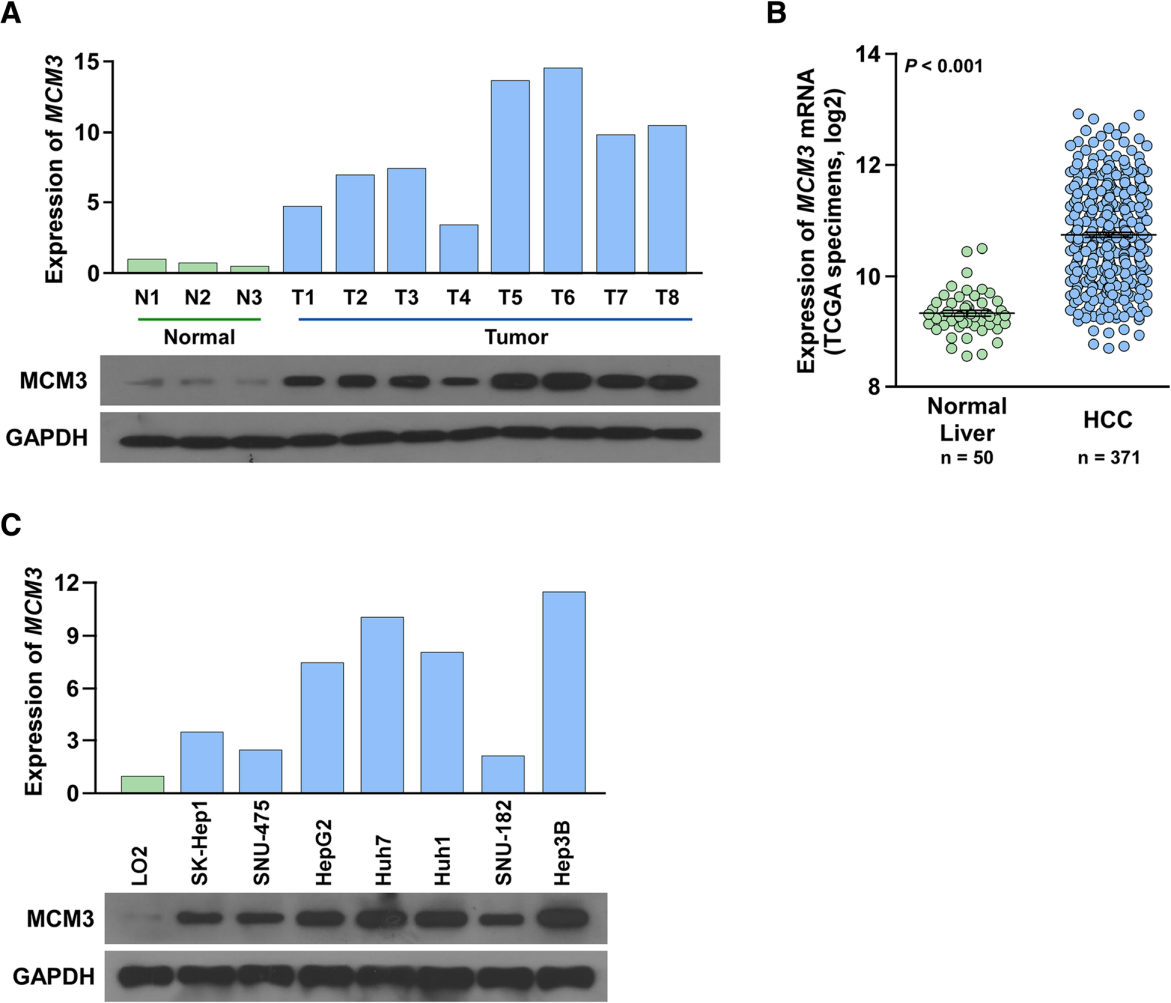
MCM3 is elevated in HCC tissues and cells. **a** qRT-PCR and western blot investigated MCM3 expression in HCC tissues and normal liver tissues. GAPDH served as an internal control. **b** Analysis of MCM3 expression in TCGA tissues. **c** qRT-PCR and western blot investigated MCM3 expression in HCC cells and immortalized normal liver cell LO2. GAPDH served as an internal control

Next, we determined MCM3 expression in HCC cells and tissues. Q-PCR and western blot analysis showed MCM3 was upregulated HCC tissues compared to normal liver tissues (Fig. 2a). We also downloaded gene expression profiles for HCC from TCGA dataset, MCM3 was significantly upregulated in HCC tissues compared to normal liver tissues (Fig. 2b). Q-PCR and western blot showed MCM3 was also upregulated in HCC cells compared to normal liver cell LO2 (Fig. 2c). These results showed that MCM3 was upregulated in HCC cells and tissues, suggesting MCM3 might promote HCC progression.

### MCM3 is associated with poor radiotherapy effect in vivo and in vitro

**Fig. 3:**
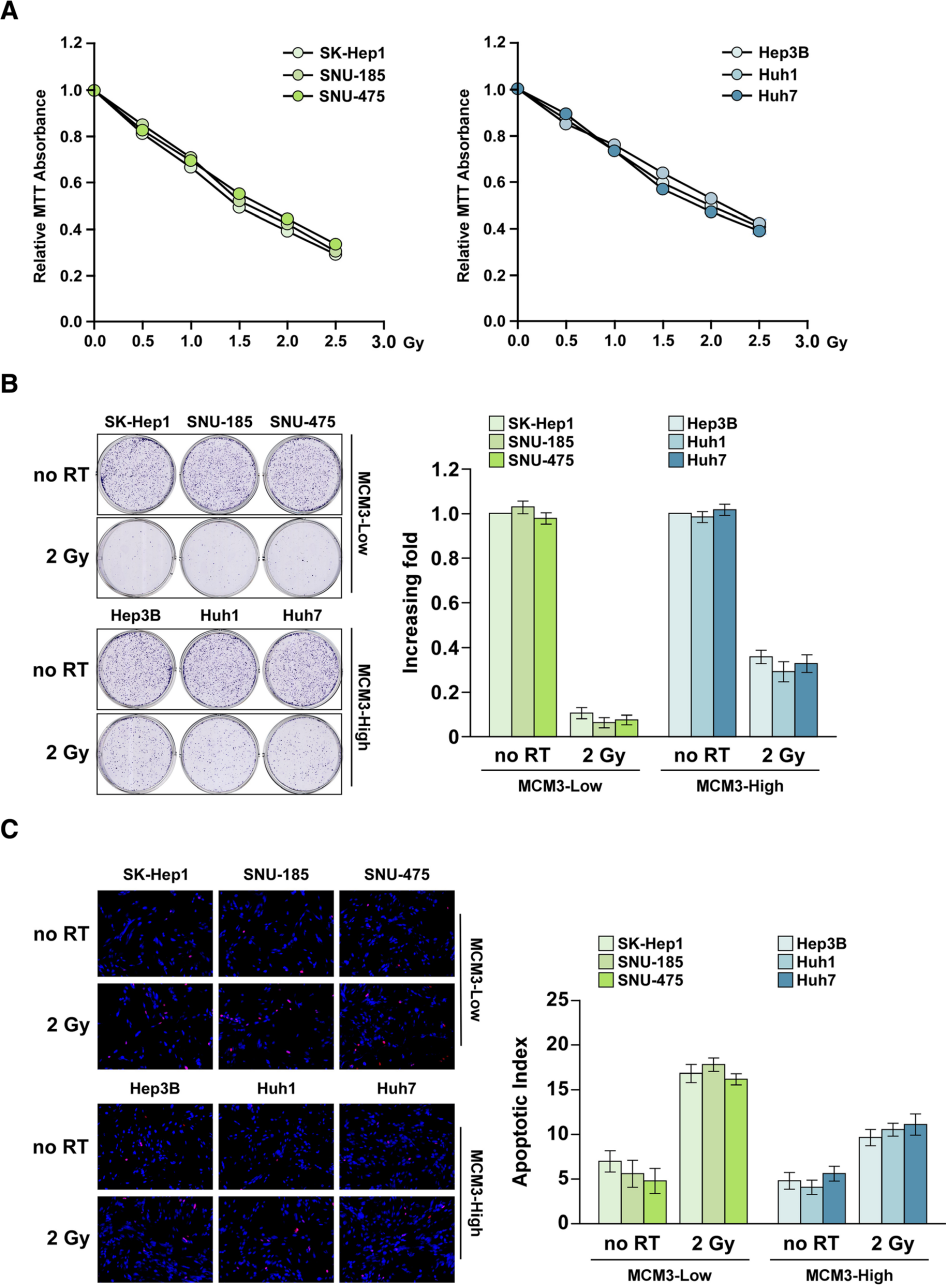
High MCM3 expression is associated with increased radiotherapy resistance. **a** MTT assay of the proliferation of HCC cell treated with different dose of radiotherapy, cells with low MCM3 expression and high MCM3 expression, respectively. **b** Colony formation of the radiotherapy effect of HCC cells with high and low MCM3 expression. **c** TUNEL assay of the radiotherapy effect of HCC cells with high and low MCM3 expression.100 μM, every experiment was independently replicated in three times. Error bars

Radiotherapy is one of the most common methods for tumor therapy, but the radioresistance is often generated after several course of treatments [28]. Some poor prognosis factors always associates with radioresistance generation, such as SRSF1 [29, 30], RPA3 [31], FOXM1 [32] and RNF6 [33], so we determined whether MCM3 regulates radioresistance. MTT analysis showed HCC cells with low MCM3 expression had low proliferation rate after radiotherapy (Fig. 3a), suggesting MCM3 might promote radioresistance. Colony formation assay showed that radiotherapy inhibit HCC cell proliferation, but the inhibition effect was better in SK-Hep1, SNU-185 and SNU-475 cells with low MCM3 expression than in Hep3B, Huh1 and Huh7 cells with high MCM expression. TUNEL assay showed that radiotherapy induced apoptosis, the induced effect was reduced in cells with high MCM3 expression, suggesting MCM3 inhibited the radiotherapy effect (Fig. 3b and c).

**Fig. 4:**
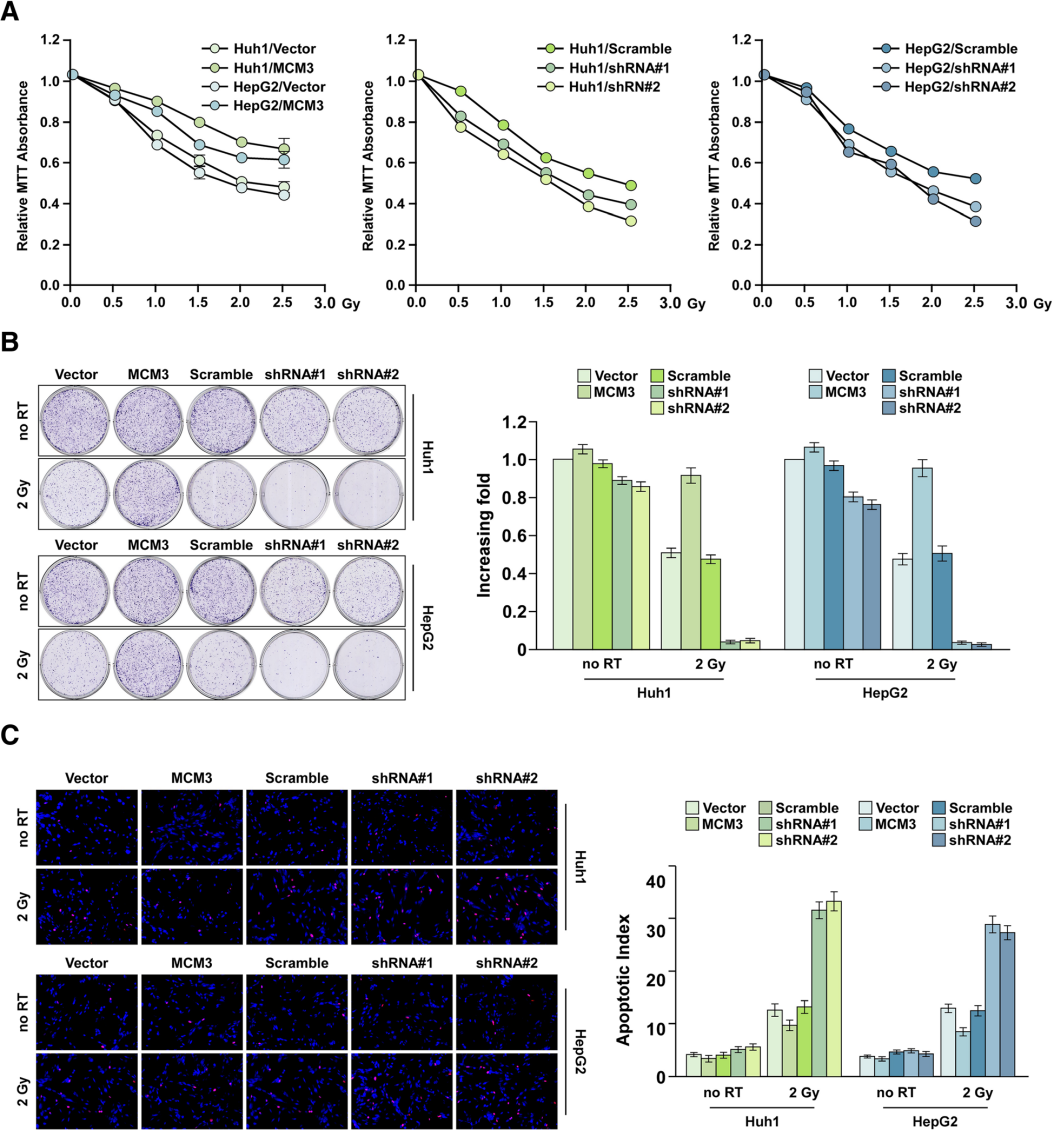
MCM3 overexpression is associated with increased radiotherapy resistance. **a** MTT assay of the proliferation of MCM3 overexpressed or knocked down HCC cell treated with different dose of radiotherapy. **b** Colony formation of the radiotherapy effect of MCM3 overexpressed or knocked down HCC cells. **c** TUNEL assay of the radiotherapy effect of MCM3 overexpressed or knocked down HCC cells. 100 μM, every experiment was independently replicated in three times. Error bars

To confirm above results, we overexpressed and knocked down MCM3 in Huh-1 and HepG2 cells, MTT assay showed that after radioresistance, the proliferation rate of cells with MCM3 overexpression was higher than control group, suggesting MCM3 overexpression increased the radioresistance, while the proliferation rate of MCM3 knockdown inhibited radioresistance (Fig. 4a). Colony formation assay showed MCM3 overexpressed inhibited radiotherapy effect, while MCM3 knockdown inhibited radioresistance. TUNEL assay showed the induced apoptosis effect was increased in cells with MCM3 knockdown compared to cells with MCM3 overexpression (Fig. 4b and c). These results suggested that MCM3 promoted radioresistance.

**Fig. 5:**
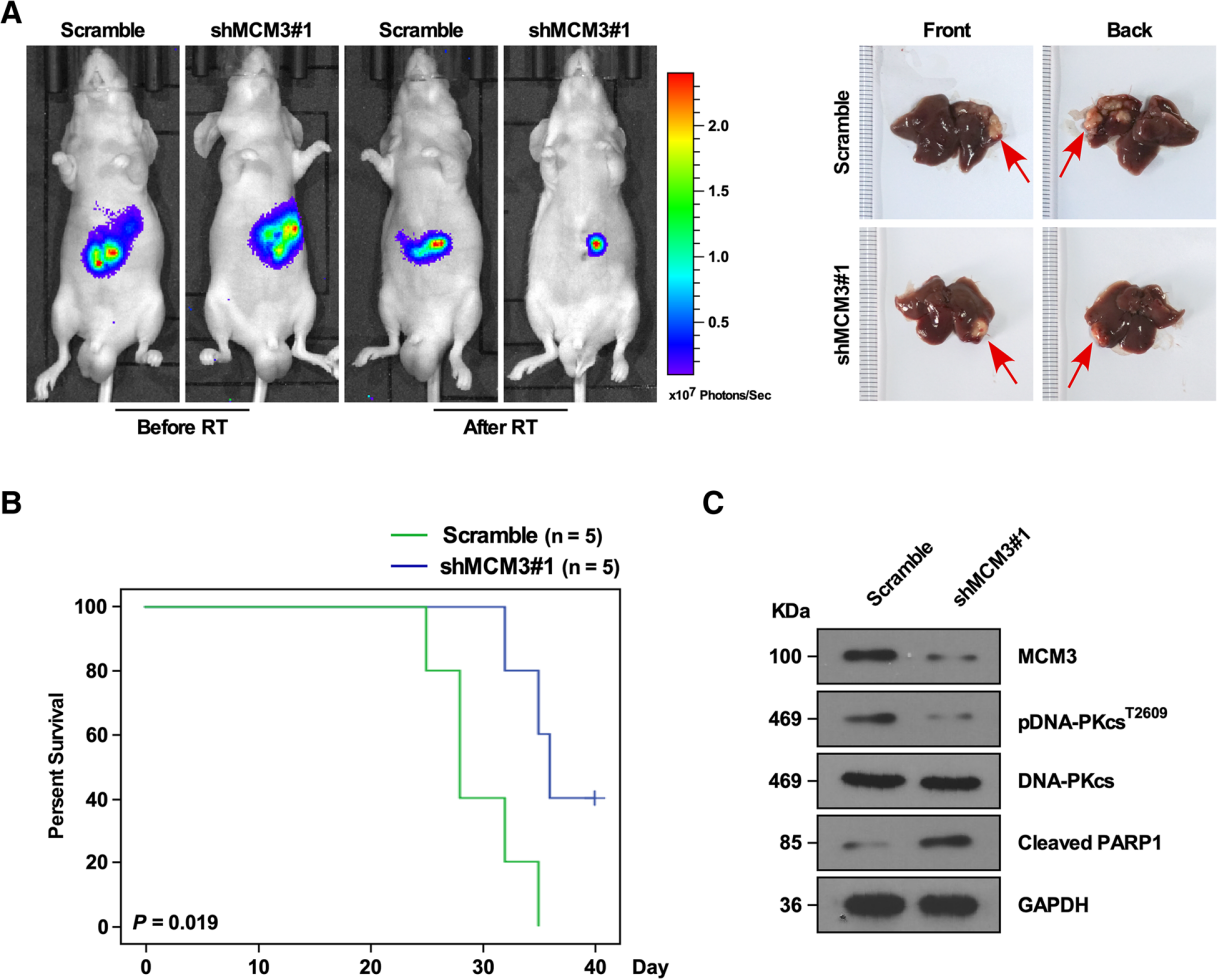
MCM3 increased radiotherapy resistance of HCC in vivo. **a** Xenograft model in nude mice treated with radiotherapy, Representative bioluminescent images of xenograft tumors formed by HepG2 cells with Scramble control and MCM3 shRNA#1, respectively (Left). and representative images of tumors in the indicated group in nude mice (Right). **b** Kaplan-Meier analysis of overall survival curves of mice with high MCM3 knockdown versus Scramble control. **c** Western blot analyzed DNA-PKcs, Pdna-PKcs^T2609^ and Cleaved PARP1. GAPDH was used as the loading control. Error bars, SD. *P < 0.05

To further confirm above findings, an in vivo model was used. MCM3 knockdown and Scramble control infected HepG2 cells with luciferase expression were injected into orthotopically injected into the liver parenchyma of nude mice, respectively. When the tumor size was up to 7.0–8.0 mm, the mice were treated with 10Gy radioactive rays. Bioluminescent images analysis showed MCM3 knockdown inhibited radioresistance, tumors were larger in Scramble groups than in MCM3 knockdown groups (Fig. 5a). Survival analysis showed mice with MCM3 knockdown had longer survival time compared to Scramble control group (Fig. 5b), suggesting MCM3 knockdown reduced radioresistance. DNA-PKcs activation is critical for development of tumor therapy resistance [34, 35], cleaved PARP1 is a marker for apoptosis [36], we isolated tumors from mice, western blot assay showed that MCM3 knockdown inhibited the phosphorylation of DNA-PKcs, and increased PARP1 cleavage (Fig. 5c), suggesting MCM3 knockdown reduced radioresistance. Together, these findings suggested that MCM3 reduced radiotherapy effect, promoted proliferation and growth, and increased anti-apoptosis ability of HCC.

### MCM3 promoted HCC radioresistance through activating NF-κB pathway

**Fig. 6:**
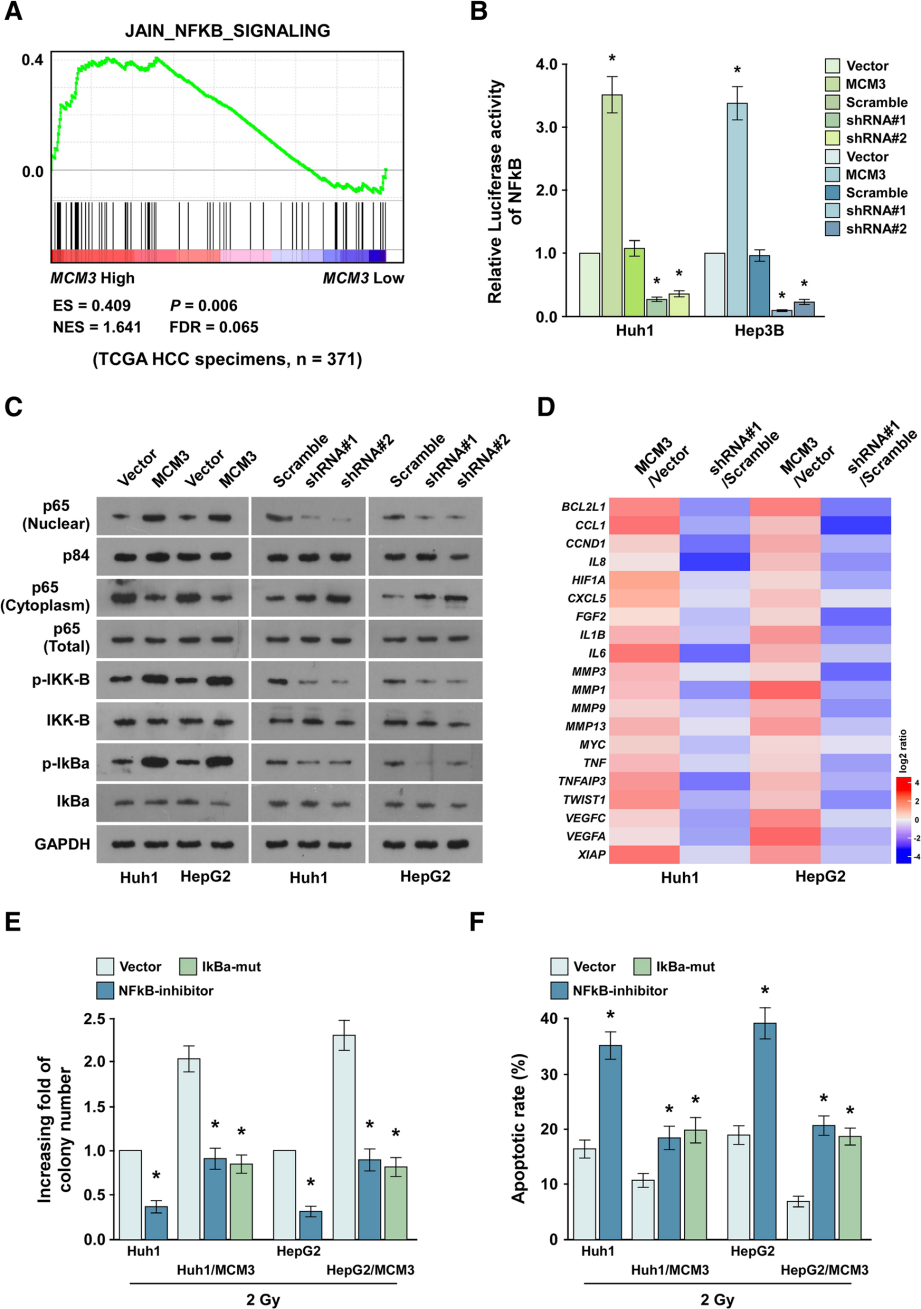
MCM3 increased radiotherapy resistance through activating NF-κB pathway. **a** GSEA revealed MCM3 expression significantly and positively correlated with TNFα induced NF-κB pathway and the upregulated target genes of NF-κB pathway. **b** Luciferase reporter assay of the effect of MCM3 overexpression or knockdown on NF-κB pathway activity. **c** Western blot analysis of p65 expression in the nuclear and cytoplasm, IKKβ and IκBα, and the phosphorylation of IKKβ and IκBα, p84 served as an internal control for nuclear proteins, GAPDH served as an internal control for total proteins. **d** qRT-PCR analysis of the expression of downstream genes of NF-κB pathway. **e** Colony formation analysis of the effect of inhibition of NF-κB pathway in MCM3 overexpression cells on radiotherapy resistance. **g** TUNEL analysis of the effect of inhibition of NF-κB pathway in MCM3 overexpression cells on radiotherapy resistance. Error bars, SD. *P < 0.05

To investigate the regulatory mechanism of MCM3 in HCC progression, we used GSEA to explore the relationship between MCM3 expression and NF-κB regulated gene signatures from the TCGA dataset, and found MCM3 was positively associated with NF-κB pathway (Fig. 6a), Luciferase assay showed the activity of the NF-κB luciferase reporter gene was significantly increased in cells overexpressing MCM3, the luciferase activity was significantly reduced in cells knocking down MCM3, suggesting MCM3 activated NF-κB pathway (Fig. 6b). The translocation of p65 into nuclear, the phosphorylation of IKK-β and IκBα is the markers of NF-κB pathway activation, western blot analysis showed that MCM3 overexpression increased the translocation of p65 to nuclear, and the phosphorylation of IKK-β and IκBα, while MCM3 knockdown inhibited the translocation of p65 to nuclear, and the phosphorylation of IKK-β and IκBα (Fig. 6c). We also analyzed the effect of MCM3 on the expression of NF-κB downstream genes [37], and found MCM3 overexpression promoted their expression, while MCM3 knockdown inhibited their expression (Fig. 6d), confirming MCM3 activated NF-κB pathway. Further confirming MCM3 activated NF-κB pathway.

**Fig. 7:**
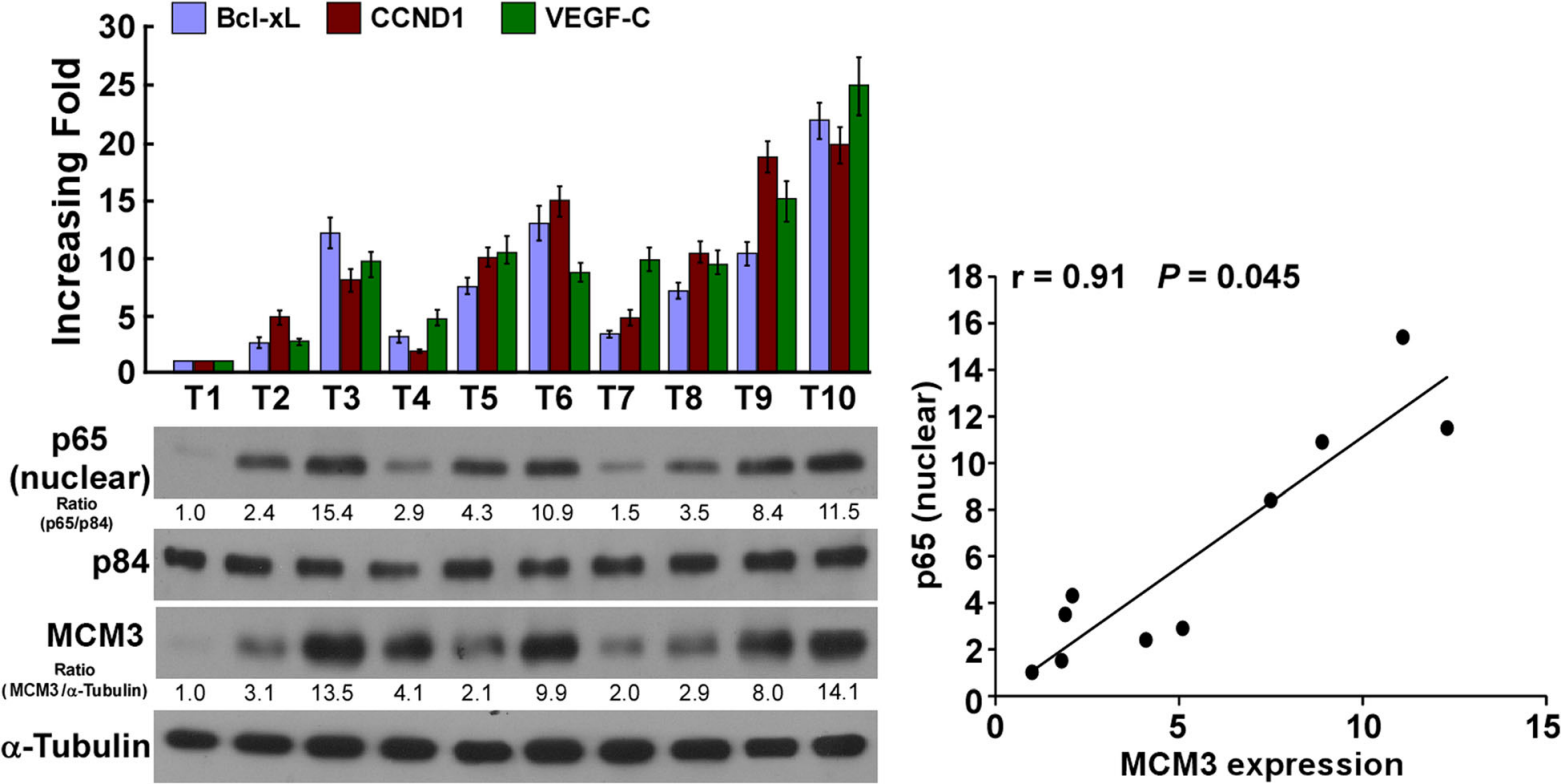
qRT-PCR analysis of CCND1, Bcl-XL and VEGF-C expression in 10 freshly collected HCC samples, western blot analysis of nuclear p65 and MCM3 expression in the same samples (Left). The correlation of nuclear p65 and MCM3 expression was showed in Right. Error bars, SD

To confirm whether MCM3 promoted radioresistance through activating NF-κB pathway, we inhibited NF-κB pathway in MCM3 overexpressing cells through adding NF-κB pathway inhibitor JSH-23 (10um) or overexpressing mutated IκBα, colony formation assay and TUNEL assay showed that inhibition of NF-κB pathway in MCM3 overexpressing cells significantly reduced radioresistance, characterized by inhibiting of cell proliferation and inducing apoptosis (Fig. 6e and f). These findings suggested MCM3 promoted HCC radioresistance through activating NF-κB pathway. We further investigated the correlation of MCM3 expression and NF-κB pathway activation in the clinic, MCM3 expression correlated with the mRNA levels of NF-κB pathway downstream genes including Bcl-xL, CCND1 and VEGF-C, and the translocation of p65 into nuclear (Fig. 7), confirming MCM3 expression related to NF-κB pathway activation in human HCC samples.

## Discussion

In present study, we found MCM3 was upregulated in HCC tissues and cells, it’s an independent prognostic factor for HCC. MCM3 overexpression increased the radioresistance, while MCM3 knockdown inhibited the radioresistance. Mechanism analysis suggested that MCM3 promoted HCC progression through activating NF-κB pathway.

We found MCM3 overexpression increased the radioresistance, previous studies show cancer stem cells are the main reason for tumor relapse, metastasis, radiotherapy and chemotherapy resistance generation [38], Many cancer types have been reported to exist cancer stem cells, including HCC, EpCAM, CD13, CD133, CD90, CD24 and CD44 have used for the markers for HCC stem cells [39, 40]. We found MCM3 increased the radioresistance of HCC, suggesting MCM3 might promote the expansion of HCC stem cells, but this inference needed to be verified by further experiments.

NF-κB pathway regulates hepatic fibrosis and HCC [41, 42], In unstimulated cells, IκB interacts with NF-κB, leading the NF-κB**/**IκB complex sequesters in the cytoplasm, and prevents NF-κB from binding to DNA. Extracellular stimuli activate NF-κB signaling, these stimuli are recognized by receptors and transmitted into the cell, where adaptor signaling proteins initiate a signaling cascade. These signaling cascades activate IKK, IKK phosphorylates IκB in the cytoplasm, leading the degradation of IκB by the proteasome and releases NF-κB from the inhibitory complex. Then NF-κB proteins trans-locates into nucleus where they bind to their target sequences and activate gene transcription [43]. We found MCM3 increased the nuclear translocation of p65 and the phosphorylation of IKK-β and IκBα, suggesting MCM3 activated NF-κB pathway. We also inhibited NF-κB pathway in MCM3 overexpressing cells, and found the radiotherapy resistance was reduced, suggesting MCM3 increased radioresistance through activating NF-κB pathway.

Although other subunits of MCM2–7 complex have been studied in tumors, such as MCM6 and MCM7, previous reporters only show MCM3 is a prognostic factor for various tumors, its function in tumor progression is reported rarely, especially in radioresistance generation, we first systematically studied the role of MCM3 in HCC radioresistance and the regulatory mechanisms. In summary, we found MCM3 increased the radiotherapy resistance of HCC through activating NF-κB pathway.

## Conclusions

In conclusion, the present study demonstrates the role of MCM3 in HCC patients’ prognosis and radioresistance, we found MCM3 was an independent prognosis factor for HCC, it promoted radioresistance of HCC through activating NF-κB pathway. Thus, MCM3 could serve as a potential biomarker for HCC prognosis and a new target for HCC therapy.

## Additional file

Additional file 1: **Table S1.** Clinicopathological characteristics of HCC patient samples. (DOCX 16 kb)

## Data Availability

The datasets supporting the conclusions of this article are included and indicated within the article.

## Abbreviations

GSEA: Gene set enrichment analysis
HCC: Hepatocellular carcinoma
MCM3: Minichromosome Maintenance 3
