# Correction to: Minichromosome maintenance 3 promotes hepatocellular carcinoma radioresistance by activating the NF-κB pathway

**DOI:** 10.1186/s13046-019-1338-1

**Published:** 2019-08-02

**Authors:** Qing Yang, Binhui Xie, Hui Tang, Wei Meng, Changchang Jia, Xiaomei Zhang, Yi Zhang, Jianwen Zhang, Heping Li, Binsheng Fu

**Affiliations:** 1 Department of Hepatic Surgery and Liver transplantation Center of the Third Affiliated Hospital, Organ Transplantation Institute, Sun Yat-sen University, Organ Transplantation Research Center of Guangdong Province, 600# Tianhe Road, Guangzhou, 510630 China; 2 Department of Hepatobiliary Surgery, The First Affiliated Hospital of Gannan Medical University, Ganzhou, 341000 China; 3 Cell-gene Therapy Translational Medicine Research Center, The Third Affiliated Hospital of Sun Yat-sen University, Guangzhou, 510630 China; 4 Guangdong Key Laboratory of Liver Disease Research, The Third Affiliated Hospital of Sun Yat-sen University, Guangzhou, 510630 China; 5 Department of Medical Oncology of the Eastern Hospital, The First Affiliated Hospital of Sun Yat-sen University, Zhongshan Er Road, Guangzhou, 510080 China

**Correction to: J Exp Clin Cancer Res**

**https://doi.org/10.1186/s13046-019-1241-9**

In the original publication of this article [1], the authors reported the order of the authors was incorrect and needs to be revised. The original article has been updated to rectify this error.

Incorrect version:

Wei Meng1, Binhui Xie2†, Qing Yang1†, Changchang Jia3, Hui Tang1†, Xiaomei Zhang4, Yi Zhang1, Jianwen Zhang1*, Heping Li5* and Binsheng Fu1*

Correct version:

Qing Yang1†, Binhui Xie2†, Hui Tang1†, Wei Meng1, Changchang Jia3, Xiaomei Zhang4, Yi Zhang1, Jianwen Zhang1*,Heping Li5* and Binsheng Fu1*
