# Correction to: Minichromosome maintenance 3 promotes hepatocellular carcinoma radioresistance by activating the NF-κB pathway

**DOI:** 10.1186/s13046-019-1395-5

**Published:** 2019-09-05

**Authors:** Qing Yang, Binhui Xie, Hui Tang, Wei Meng, Changchang Jia, Xiaomei Zhang, Yi Zhang, Jianwen Zhang, Heping Li, Binsheng Fu

**Affiliations:** 1 Department of Hepatic Surgery and Liver transplantation Center of the Third Affiliated Hospital, Organ Transplantation Institute, Sun Yat-sen University; Organ Transplantation Research Center of Guangdong Province, 600# Tianhe Road, Guangzhou, 510630 China; 2 Department of Hepatobiliary Surgery, The First Affiliated Hospital of Gannan MedicalUniversity, Ganzhou, 341000 China; 3 Cell-gene Therapy Translational Medicine Research Center, The Third Affiliated Hospital of Sun Yat-sen University, Guangzhou, 510630 China; 4 Guangdong Key Laboratory of Liver Disease Research, The Third Affiliated Hospital of Sun Yat-sen University, Guangzhou, 510630 China; 5 Department of Medical Oncology of the Eastern Hospital, The First Affiliated Hospital of Sun Yat-sen University, Zhongshan Er Road, Guangzhou, 510080 China

**Correction to: J Exp Clin Cancer Res**

**https://doi.org/10.1186/s13046-019-1241-9**

In the original publication of this article [1], the Fig. 7 is wrong, but does not affect discussions and conclusions drawn in the article.

The corrected Fig. 7 is shown below:

**Fig. 7:**
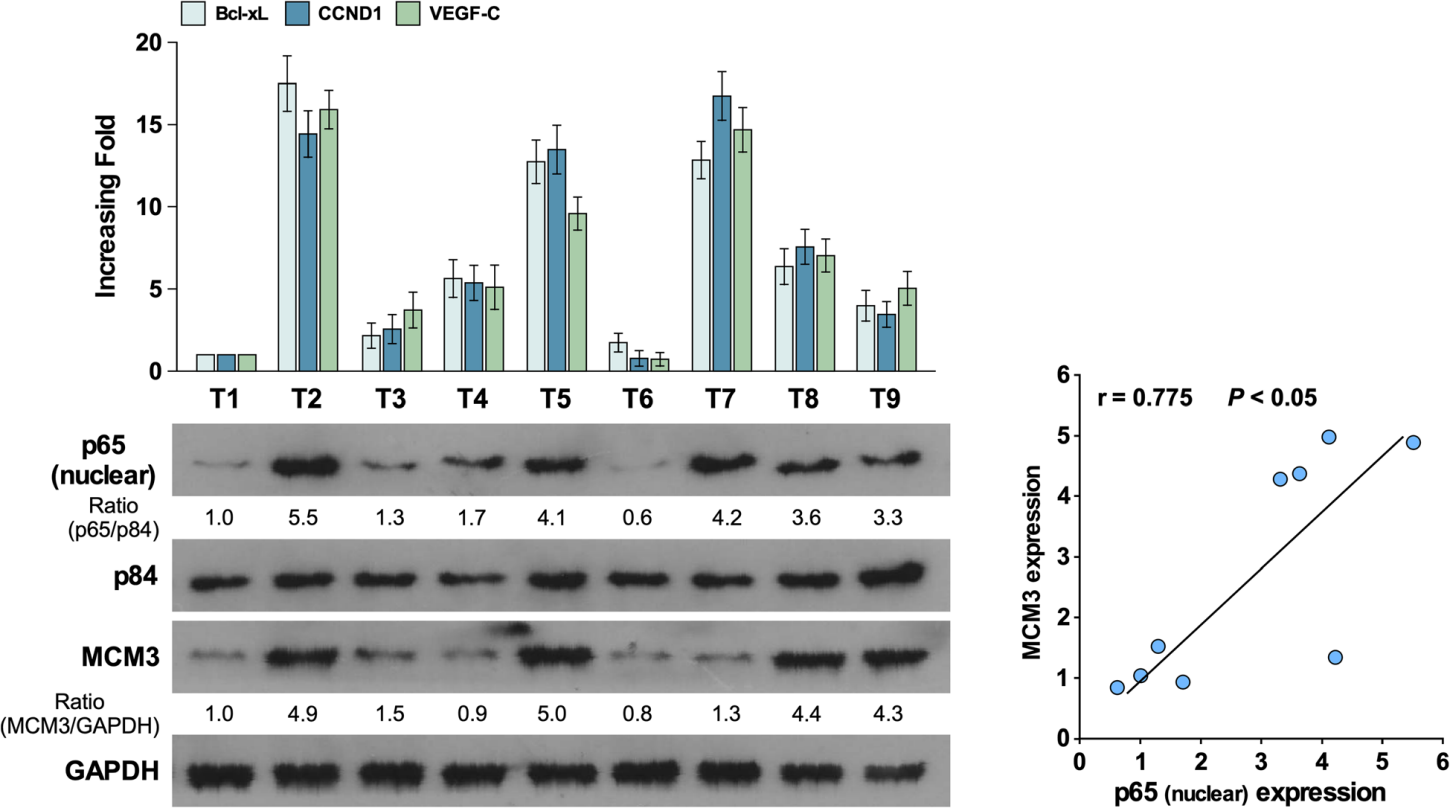
qRT-PCR analysis of CCND1, Bcl-XL and VEGF-C expression in 10 freshly collected HCC samples, western blot analysis of nuclear p65 and MCM3 expression in the same samples (Left). The correlation of nuclear p65 and MCM3 expression was showed in Right. Error bars, SD
