# Supplementary material for: Minichromosome maintenance 3 promotes hepatocellular carcinoma radioresistance by activating the NF-κB pathway
Source: J Exp Clin Cancer Res. 2019 Jun 17;38:263. doi: 10.1186/s13046-019-1241-9 (PMC6580494; doi:10.1186/s13046-019-1241-9)
Supplement: Supplementary file 1 — Table S1. Clinicopathological characteristics of HCC patient samples. (DOCX 16 kb) [file 13046_2019_1241_MOESM1_ESM.docx]

**Additional file 1: Table S1 Clinicopathological characteristics of HCC patient samples**

|  | **Number of cases** |
| --- | --- |
| **Gender** |  |
| Male | 152 |
| Female | 10 |
| **Age(years)** |  |
| > 45 | 109 |
| ≤ 45 | 53 |
| **BCLC** |  |
| 0 | 10 |
| 1 | 123 |
| 2 | 13 |
| 3 | 16 |
| **MVI** |  |
| 0 | 2 |
| 1 | 8 |
| 2 | 152 |
| **Chemotherapy** |  |
| Yes | 6 |
| No | 156 |
| **Relapse** |  |
| Yes | 119 |
| No | 43 |
| **Pathologic Differentiation** |  |
| Well | 30 |
| Moderate | 117 |
| Poor | 15 |
| **Cirrhosis** |  |
| Yes | 42 |
| No | 120 |
| **HBsAg** |  |
| Yes | 143 |
| No | 19 |
| **Drinking** |  |
| Yes | 52 |
| No | 110 |
| **Smoking** |  |
| Yes | 78 |
| No | 84 |
| **Survival** |  |
| Yes | 119 |
| No | 43 |
| **MCM3** |  |
| High level | 77 |
| Low level | 85 |
